# Supplementary material for: Resolving the full spectrum of human genome variation using Linked-Reads
Source: Genome Res. 2019 Apr;29(4):635–45. doi: 10.1101/gr.234443.118 (PMC6442396; doi:10.1101/gr.234443.118)
Supplement: Supplemental Material [file supp_gr.234443.118_Supplemental_longranger-2.2.zip › longranger-2.2.tar.bz2!/lr2.2-public/lib/stan/cmdstan/stan/doc/api/html/agrad_2fwd_2functions_2binomial__coefficient__log_8hpp_source.html]

Stan: src/stan/agrad/fwd/functions/binomial\_coefficient\_log.hpp Source File


|  |  |
| --- | --- |
|  | Stan  2.5.0  probability, sampling & optimization |


- Main Page
- Related Pages
- Namespaces
- Classes
- Files

- File List
- File Members

All Classes Namespaces Files Functions Variables Typedefs Enumerations Enumerator Friends Macros Pages

- src
- stan
- agrad
- fwd
- functions

binomial\_coefficient\_log.hpp

Go to the documentation of this file.

1 #ifndef STAN\_\_AGRAD\_\_FWD\_\_FUNCTIONS\_\_BINOMIAL\_COEFFICIENT\_LOG\_HPP

2 #define STAN\_\_AGRAD\_\_FWD\_\_FUNCTIONS\_\_BINOMIAL\_COEFFICIENT\_LOG\_HPP

3

4 #include <stan/agrad/fwd/fvar.hpp>

5 #include <stan/meta/traits.hpp>

6 #include <boost/math/special\_functions/digamma.hpp>

7 #include <stan/math/functions/binomial\_coefficient\_log.hpp>

8

9 namespace stan {

10

11  namespace agrad {

12

13  template <typename T>

14  inline

15  fvar<T>

16  binomial\_coefficient\_log(const fvar<T>& x1, const fvar<T>& x2) {

17  using boost::math::digamma;

18  using std::log;

19  using stan::math::binomial\_coefficient\_log;

20  const double cutoff = 1000;

21  if ((x1.val\_ < cutoff) || (x1.val\_ - x2.val\_ < cutoff)) {

22  return fvar<T>(binomial\_coefficient\_log(x1.val\_, x2.val\_),

23  x1.d\_ \* digamma(x1.val\_ + 1)

24  - x2.d\_ \* digamma(x2.val\_ + 1)

25  - (x1.d\_ - x2.d\_) \* digamma(x1.val\_ - x2.val\_ + 1));

26  } else {

27  return fvar<T>(binomial\_coefficient\_log(x1.val\_, x2.val\_),

28  x2.d\_ \* log(x1.val\_ - x2.val\_)

29  + x2.val\_ \* (x1.d\_ - x2.d\_) / (x1.val\_ - x2.val\_)

30  + x1.d\_ \* log(x1.val\_ / (x1.val\_ - x2.val\_))

31  + (x1.val\_ + 0.5) / (x1.val\_ / (x1.val\_ - x2.val\_))

32  \* (x1.d\_ \* (x1.val\_ - x2.val\_) - (x1.d\_ - x2.d\_) \* x1.val\_)

33  / ((x1.val\_ - x2.val\_) \* (x1.val\_ - x2.val\_))

34  - x1.d\_ / (12.0 \* x1.val\_ \* x1.val\_)

35  - x2.d\_

36  + (x1.d\_ - x2.d\_) / (12.0 \* (x1.val\_ - x2.val\_)

37  \* (x1.val\_ - x2.val\_))

38  - digamma(x2.val\_ + 1) \* x2.d\_);

39  }

40  }

41

42  template <typename T>

43  inline

44  fvar<T>

45  binomial\_coefficient\_log(const fvar<T>& x1, const double x2) {

46  using boost::math::digamma;

47  using std::log;

48  using stan::math::binomial\_coefficient\_log;

49  const double cutoff = 1000;

50  if ((x1.val\_ < cutoff) || (x1.val\_ - x2 < cutoff)) {

51  return fvar<T>(binomial\_coefficient\_log(x1.val\_, x2),

52  x1.d\_ \* digamma(x1.val\_ + 1)

53  - x1.d\_ \* digamma(x1.val\_ - x2 + 1));

54  } else {

55  return fvar<T>(binomial\_coefficient\_log(x1.val\_, x2),

56  x2 \* x1.d\_ / (x1.val\_ - x2)

57  + x1.d\_ \* log(x1.val\_ / (x1.val\_ - x2))

58  + (x1.val\_ + 0.5) / (x1.val\_ / (x1.val\_ - x2))

59  \* (x1.d\_ \* (x1.val\_ - x2) - x1.d\_ \* x1.val\_)

60  / ((x1.val\_ - x2) \* (x1.val\_ - x2))

61  - x1.d\_ / (12.0 \* x1.val\_ \* x1.val\_)

62  + x1.d\_ / (12.0 \* (x1.val\_ - x2) \* (x1.val\_ - x2)));

63  }

64  }

65

66  template <typename T>

67  inline

68  fvar<T>

69  binomial\_coefficient\_log(const double x1, const fvar<T>& x2) {

70  using boost::math::digamma;

71  using std::log;

72  using stan::math::binomial\_coefficient\_log;

73  const double cutoff = 1000;

74  if ((x1 < cutoff) || (x1 - x2.val\_ < cutoff)) {

75  return fvar<T>(binomial\_coefficient\_log(x1, x2.val\_),

76  -x2.d\_ \* digamma(x2.val\_ + 1)

77  - x2.d\_ \* digamma(x1 - x2.val\_ + 1));

78  } else {

79  return fvar<T>(binomial\_coefficient\_log(x1, x2.val\_),

80  x2.d\_ \* log(x1 - x2.val\_)

81  + x2.val\_ \* -x2.d\_ / (x1 - x2.val\_)

82  - x2.d\_

83  - x2.d\_ / (12.0 \* (x1 - x2.val\_) \* (x1 - x2.val\_))

84  + x2.d\_ \* (x1 + 0.5) / (x1 - x2.val\_)

85  - digamma(x2.val\_ + 1) \* x2.d\_);

86  }

87  }

88  }

89 }

90 #endif

stan::agrad::fvar

**Definition:** fvar.hpp:13

stan::math::binomial\_coefficient\_log

boost::math::tools::promote\_args< T\_N, T\_n >::type binomial\_coefficient\_log(const T\_N N, const T\_n n)

Return the log of the binomial coefficient for the specified arguments.

**Definition:** binomial\_coefficient\_log.hpp:63

traits.hpp

fvar.hpp

stan::agrad::binomial\_coefficient\_log

fvar< T > binomial\_coefficient\_log(const fvar< T > &x1, const fvar< T > &x2)

**Definition:** binomial\_coefficient\_log.hpp:16

stan::agrad::digamma

fvar< T > digamma(const fvar< T > &x)

**Definition:** digamma.hpp:16

binomial\_coefficient\_log.hpp

stan::agrad::fvar::d\_

T d\_

**Definition:** fvar.hpp:16

stan::agrad::log

fvar< T > log(const fvar< T > &x)

**Definition:** log.hpp:15

stan::agrad::fvar::val\_

T val\_

**Definition:** fvar.hpp:15


---

[ Stan Home Page ]

*© 2011–2014,
Stan Development Team.*
